# Video Capsule Endoscopy in the Assessment of Portal Hypertensive Enteropathy

**DOI:** 10.1155/2018/5109689

**Published:** 2018-11-01

**Authors:** Yasir Al-Azzawi, Lidia Spaho, Mohammed Mahmoud, Joan Kheder, Anne Foley, David Cave

**Affiliations:** ^1^University of Massachusetts School of Medicine, 55 North Lake Ave, Worcester, MA 01606, USA; ^2^University of Massachusetts School of Medicine, USA

## Abstract

**Background:**

The features of the portal hypertension enteropathy (PHE) vary from mild mucosal changes to varices with or without bleeding. The prevalence and the development are not fully understood.

**Aim:**

Our aim is to examine the prevalence and the different manifestations of PHE using video capsule endoscopy (VCE).

**Methods:**

It is a single center retrospective study of patients with cirrhosis, who had VCE. Based on the published literature, we divided the PHE lesions into vascular lesions and mucosal lesions.

**Results:**

Of the 100 patients with cirrhosis that had a VCE study, the mean age was 62.82 years. Male gender was predominant (64%), while Caucasians represented 82% of the cohort. The most common etiology of cirrhosis was chronic alcohol abuse followed by chronic hepatitis C virus and nonalcoholic steatohepatitis. VCE detected small bowel lesions in 71% of the patients while the features of PHE were found in 65% from the total cohort. AVMs and inflammatory changes were the most common findings, followed by bleeding. More than 50% of the lesions were vascular in nature. The risk of finding PHE in decompensated cirrhosis is twice that in compensated cirrhosis. Forty-five patients had negative EGD exam for any active bleeding, esophageal varices, portal hypertensive gastropathy, or gastric varices. Of these, 69% had features of PHE in their VCE.

**Conclusions:**

VCE detected small bowel lesions in 71% of our cohort. There is a high prevalence of PHE in decompensated cirrhosis. Vascular lesions are the most common finding in the small bowel of this population.

## 1. Introduction

Cirrhosis or end-stage liver disease is defined as a late stage of scarring and fibrosis of the liver and characterized by alteration of the hepatic architecture as well as the development of regenerative nodules; these changes can lead to a multitude of complications. Cirrhosis can be most commonly attributed to excessive alcohol consumption, viral hepatitis, or nonalcoholic fatty liver disease [1]. Reports from the Centers for Disease Control (CDC) estimate that 75,766 deaths and 2.3 million years of potential life lost during 2001 were attributable to excessive alcohol abuse, which also contributed to 60-70% of the cirrhosis in the USA. It is the 12^th^ leading cause of death in USA and it is the leading cause of morbidity [2]. A recent population-based study showed that, per 2-year interval, cirrhotic patients have 26.4% mortality rate as compared to propensity matched controls who had a mortality rate of 8.4% [3].

The pathophysiology behind developing portal hypertension (PH) is believed to be related to scarring in the liver parenchymal disease, the increased production of vasoconstrictor (endothelins, angiotensin II, norepinephrine, and thromboxane A2), and a decrease in secretion of endothelial vasodilators. As a result, dysregulation of the extrahepatic vascular beds occurs in the splanchnic and systemic circulations causing the blood flow inside the portal veins to increase and the portal vein pressure to rise. Approximately 50% of the patients develop esophageal varices while a small percentage develops complications in the other part of the gastrointestinal tract like ectopic varices in the small bowel [4, 5].

With the availability of VCE in the last two decades, the evaluation of the small bowel has become easy. The ramifications of PH in the small bowel are referred to as portal hypertensive enteropathy (PHE). Multiple studies have attempted to define PHE and identify the major components that it encompasses. Originally in 2005, De Palma et al. had noted that PHE included mucosal inflammatory changes such as edema, erythema, granularity, friability, and spontaneous bleeding as well as vascular lesions such as cherry red spots, telangiectasias, angiodysplasia, and varices [6]. Later in 2008, Kodama et al. used double balloon enteroscopy to classify PHE into villous lesions and vascular lesions [7]. Despite the classifications, there is no gold standard scoring system to differentiate the severity of endoscopic abnormalities found [8].

With the current definitions as stated above, the prevalence of PHE has been noted to vary between 18% and 100% in patients with cirrhosis. The majority of data, when using a broad definition of PHE, indicates that prevalence is >60%. However, not all patients with cirrhosis have PHE; but there are several risk factors that increase the likelihood of PHE: CTP B and C, portosystemic shunts, ascites, portal thrombosis, esophageal varices, and portal hypertensive gastropathy [6, 9].

These patients are at a high risk of GI bleeding, especially small intestinal bleeding that cannot be identified by EGD and colonoscopies. Video capsule endoscopy has been used more frequently in the last decade to identify the bleeding source. VCE serves as the road map to allow for further interventions that need to be taken to evaluate and treat gastrointestinal bleeding.

## 2. Materials and Methods

We retrospectively reviewed the medical record of 100 consecutive patients who were found to be cirrhotic and to have complete video capsule studies between 2010 and 2016 at the University of Massachusetts, UMass Medical Center. Approval was obtained from the University of Massachusetts Institutional Review Board (IRB). By using the ICD codes of cirrhosis and the procedure code of the video capsule endoscope, we were able to identify our patient's cohort. The chart of each patient was reviewed, and the extracted data included the general demographic features of the cohort: the age, gender, and the race of the patients. The diagnosis of cirrhosis was made by histological findings or compatible typical radiographic features, clinical presentations, and laboratory tests. The severity of cirrhosis was classified using two classifications: Child-Pugh classification and the model for end-stage liver disease (MELD) score classification.

Two generations of Given Imaging capsules were used during the study period including the consecutive generations of PillCam SB2 and SB3 (Medtronic, Minneapolis, MN). Reading software was Rapid v 6-8. Video capsule endoscopy studies were read by experienced gastroenterologists and the findings were documented in in Provation (Provation, Minneapolis, MN). The findings used in this study included the indications for the video capsule evaluation as documented in the charts of the patients which were obscure GI bleed, anemia, and/or abnormal findings on the cross-sectional imaging studies.

## 3. Classification

Our classification of the PHE was based on the published studies reported by De Palma et al., Kodama et al., and Abdelaal et al. [6, 7, 10]. The first classification of the PHE was reported by De Palma et al. in 2005. We divided PHE into two grades: Grade 1 which includes edema, erythema, granularity, friability, and/or spontaneous bleeding and Grade 2 which includes cherry-red spots, telangiectasias, angiodysplasia-like lesions, and varices.

Kodama et al. divided PHE into villous lesions and vascular lesions. Villous lesions include either edema, atrophy, or erythematous changes of the villi while vascular lesions include varices, red spots, vascular spiders, and lymphoid follicles with dilated vessels and dilated/proliferated vessels. Lastly, Abdelaal et al. diagnosed PHE based on his scoring system which depended on four main types of mucosal lesions of the small intestine: red spots, angioectasias, varices, and inflammatory-like lesions.

In our study, we divided the PHE lesions into two types of lesions: vascular and mucosal lesions. Vascular lesions included arteriovenous malformation (AVM), red spots, bleeding, or varices while the mucosal lesions included mild inflammatory changes such as erythematous changes and edema or severe inflammatory changes including mosaic-like pattern or snakeskin mucosal changes, and congested and friable mucosa.

We also divided our cohort based on their Child-Turcotte-Pugh (CTP) classification into classes A, B, and C using the patients' clinical presentations of ascites and hepatic encephalopathy, in addition to their laboratory findings.

## 4. Statistical Analysis

Comparisons were performed using the unpaired chi-square test for quantitative data and the chi-squared test for categorical data. A Yates correction or Fisher's exact test was used when required. P-value < 0.05 was considered statistically significant. Odds ratios (ORs) and 95% confidence intervals (95% CIs) were used in the analyses.

## 5. Results

**Figure 1:**
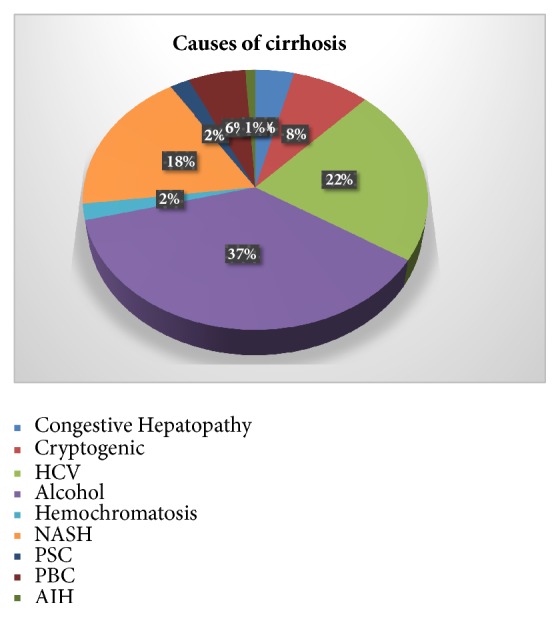
Causes of cirrhosis. HCV: hepatitis C virus, NASH: nonalcoholic steatohepatitis, PSC: primary sclerosing cholangitis, PBC: primary biliary cirrhosis, and AIH: autoimmune hepatitis.

One hundred patients with cirrhosis had a VCE study between 2010 and 2016. The mean age of the cohort was 62.82±2 years and the mean/MELD score was 13.86±0.66. Male gender was predominant with 64% of the cohort while the Caucasians represented 82% (Table 1). The most common etiologies of the cirrhosis in our cohort were chronic alcohol abuse followed by chronic hepatitis C virus (HCV) and nonalcoholic steatohepatitis (NASH) with percentages of 37%, 22%, and 18%, respectively (Figure 1).

**Figure 2:**
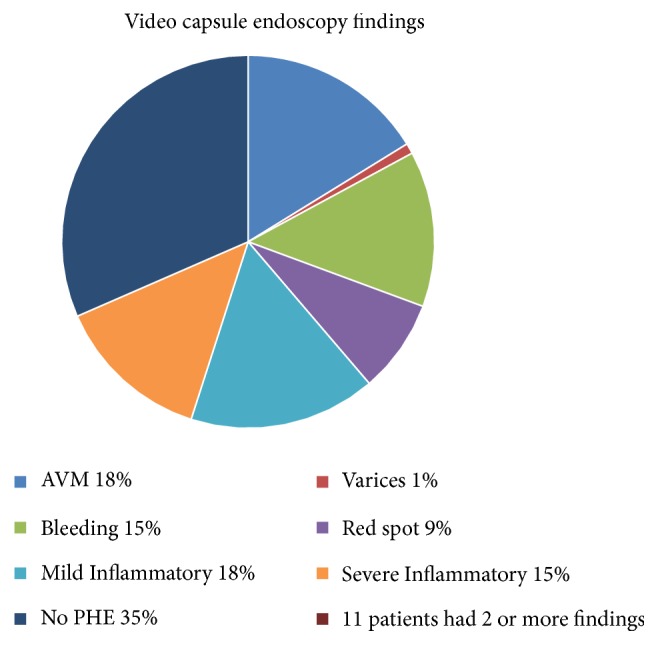
Video capsule endoscopy findings.

Small intestine evaluation by VCE detected small intestinal lesions in 71% of the patients while 29% patients had normal study. The features of PHE were found in 65% of the cohort. Vascular lesions were found in 43% of the cohort and inflammatory lesions were found in 33%. 11 patients were found to have more than one type of PHE types with vascular lesions comprising more than 50% of the PHE lesions. AVMs and inflammatory changes were the most common findings, followed by bleeding with percentages of 18%, 18%, and 15%, respectively (Figure 2).

Our cohort had more decompensated cirrhosis defined by CTP B and C than compensated cirrhosis which is defined as CTP A. Decompensated cirrhosis (CTP B and C) represented 54% of the cohort while compensated cirrhosis (CTP A) represented 46% of the cohort.

When the two groups were compared, compensated cirrhosis versus decompensated cirrhosis, our data showed that the risk of having PHE in decompensated cirrhosis (CTP B and C) is two times the risk in compensated cirrhosis with an odd ratio of 2 and P-value of 0.01. Our study found that there was no correlation between thrombocytopenia and PHE. The risk of PHE is double that in patients with thrombocytopenia when compared with patients with normal platelet count but did not reach statistically significant value with P-value 0.09 (Table 2).

There was no correlation between the presence of PHE and patients' gender in this study. In addition, there was no correlation between the etiology of cirrhosis and PHE. We evaluated the correlation between PHE and other endoscopic features of portal hypertension like esophageal varices, portal hypertension gastropathy, and gastric varices. Of the 45 patients which had negative EGD exam for any esophageal varices (EV), portal hypertensive gastropathy (PHG), or gastric varices (GV), 31 patients (69%) had features of portal hypertension enteropathy in their VCE. 55 patients had features of portal hypertension through the EGD exam including EV, portal hypertension gastropathy, or gastric varices, and 34 of them (61%) had PHE. Our study also showed that the presence of PHE does not correlate with the presence or the absence of the EV, portal hypertension gastropathy, or gastric varices in cirrhotic patients (Table 2).

## 6. Discussion

The features of portal hypertension in the small intestine vary from normal mucosa to bleeding ectopic varices that can lead to death. It can involve any part of the gastrointestinal tract: esophageal varices, gastropathy, small intestine enteropathy, and/or colopathy [11]. Historically, the first article describing portal hypertension features in the small intestine was reported by Jonas et al. who studied those changes in rats. They described portal hypertension enteropathy as changes in the small intestinal mucosa that includes erythema, decreased duodenal villous perfusion, and an increased susceptibility to injuries [12].

The features of portal hypertension enteropathy were first described endoscopically by Thiruvengadam as an erythematous change in the small intestinal mucosa that resembled the features of gastropathy and both changes had been called portal hypertension gastroenteropathy [13]. The introduction of video capsule endoscopy and small intestine enteroscopy devices helped in better estimating the prevalence of PHE. More data was published in the last decade describing the features of the portal hypertension enteropathy (PHE). Most of these published reports as mentioned above classified the PHE into two categories: vascular lesions (arteriovenous malformation (AVM), red spots, bleeding, and varices) and inflammatory lesions (mild: erosions, erythema or severe: edema, congested, granular, and friable mucosa) [6, 7, 10].

Most of the information that we have about PHE is from relatively small size studies, both retrospective and prospective. These studies estimated the prevalence to have a wide range between 18% and 100% but the majority of these studies specially the one with large sample size have a narrower range of 65% to 69% [8]. Our cohort is the largest cohort study in the USA including 100 cirrhotic patients showing that the prevalence of the PHE is 65%, which is similar to the previously published reports [5–8]. Vascular lesions were detected more than the inflammatory lesions with percentages of 43% and 33%, respectively.

We found no association between the PHE and the age, gender, and the etiology of cirrhosis which is similar to the findings of other published studies. The association of PHE and the presence of esophageal varices and/or portal hypertension gastropathy was documented by both Goulas et al. and Aoyama et al. [11, 14]. This association was found to be in accordance with a study reported by De Palma et al., which showed a strong association between the portal hypertension enteropathy with larger esophageal varices, portal hypertension, gastropathy, and colopathy.

In our study, 45 patients had a negative EGD study for any active bleeding or signs of portal hypertension: esophageal varices, gastric varices, or portal hypertension gastropathy. Thirty-one of them (67%) had features of portal hypertension enteropathy in their VCE evaluation of the small intestine. Therefore, no association was found between the PHE and EV, portal hypertension gastropathy, or gastric varices.

The prevalence of each type of PHE is different between the published studies. The prevalence of vascular types of PHE like AVMs, red spots, and varices ranged within 24%-55%, 22%-62%, and 8%-38%, respectively [9, 15]. In our study, the prevalence is lower than the published studies: the prevalence of the vascular AVMs, red spots, and varices was 18%, 15%, and 1%, respectively.

The reported prevalence of inflammatory lesions ranges between 5% and 13% [9, 10, 16, 17]. Our cohort had a higher prevalence of mild and severe inflammatory changes of 15-18%, respectively.

The correlation between PHE and the liver function demonstrated by Child-Turcotte-Pugh (CTP) classification is still controversial. The Child-Pugh classification system takes into consideration the synthetic function of the liver (albumin, INR, and bilirubin) and the clinical presentations of the portal hypertension (ascites and hepatic encephalopathy). Misra et al., Alessandro et al., Pennazio et al., and Goulas et al. found that there is no association between the CTP score and the presence of PHE [14, 16–18].

In contrast, Aoyama et al. found that PHE is associated with higher CTP score [11]. De Palma et al. and Abdulaal et al. both confirmed the association between PHE and the CTP score as a linear correlation [6, 10]. Our study supports that correlation as we found a strong association between the CTP score and the presence of PHE. The risk of having PHE in decompensated cirrhosis is twice the risk in the compensated cirrhosis with an odd ratio of 2.0 with CI 95% (P<0.05).

The absence of the features of portal hypertension in the esophagus and stomach did not exclude the presence of PHE. Therefore, the absence of association between PHE and portal hypertension features in the stomach and esophagus should not preclude the evaluation of the small intestine as source of bleeding in the cirrhotic patient and VCE might be warranted regardless the existence of gastroesophageal portal hypertension features.

These findings provide further insight to the role of the VCE that can assess in management of these populations, though our study has its limitation, being a single center, being retrospective in design, and including a high percentage of Caucasians, but it is the first to evaluate such findings in cirrhotic patients.

## 7. Conclusion

We conclude that one-third of cirrhotic patients with suspect GI bleed/anemia have treatable PHE lesions. AVMs, varices, and bleeding lesions are all treatable lesions that can be evaluated by video capsule endoscopy and treated by small bowel enteroscopy, single balloon enteroscopy, double balloon enteroscopy, or trans-jugular intrahepatic-portosystemic shunting. Hepatologists and gastroenterologists should not be hesitant to pursue this investigative modality in this population, when no active bleeding is found by traditional EGD and colonoscopic evaluation.

## 8. Summary

Portal hypertension enteropathy is prevalent in cirrhotic patient and carries the risk of obscure GI bleeding. The published data regards the small samples sizes which suggested the prevalence, and our cohort, the largest cohort in the US, supported the previous facts. Our data supports the fact that PHE was associated with decompensated cirrhosis. The absence of varices in the stomach or esophagus should not distract the gastroenterologist from further investigating the small intestine for features of portal hypertension.

## Figures and Tables

**Table 1:** Demographic MELD: model for end-stage liver disease.

Demographic	Number & (%)
Total number of patients	100

Age	62.82±2

MELD score	13.86(±0.66)

Demographics:	

Male	64(64%)

Female	36(36%)

Whites	82(82%)

Asian	2(2%)

African-American	1(1%)

Others	15(15%)

**Table 2:** The predictor factors of PHE. CTP-A: Child-Turcotte-Pugh score A, CTP-B+C: Child-Turcotte-Pugh score B and C, HCV: hepatitis C virus, NASH: nonalcoholic steatohepatitis, PSC: primary sclerosing cholangitis, PBC: primary biliary cirrhosis, EV: esophageal varices, PHG: portal hypertension gastropathy, GV: gastric varices, PHE: portal hypertension enteropathy, and OR: odd ratio.

Predictor factors	Number	PHE	No PHE	OR	P-value
CTP-A	46	26	20	0.5	0.01

CTP-B+C	54	39	15	2.0	0.01

Alcohol	37	20	17	0.4	0.04

HCV	22	13	9	0.4	0.02

NASH	18	14	4	2.1	0.1

Cholestatic liver disease (PSC+PBC)	8	6	2	1.6	0.4

Thrombocytopenia	69	48	21	1.8	0.09

Male	64	42	22	1.1	0.7

Female	36	23	13	0.9	0.7

EGD + (EV, PHG, or GV)	55	34	21	0.7	0.5

EGD -	45	31	14	1.3	0.5

## Data Availability

The data used to support the findings of this study are available from the corresponding author upon request.
